# WNK3 promotes the invasiveness of glioma cell lines under hypoxia by inducing the epithelial-to-mesenchymal transition

**DOI:** 10.1515/tnsci-2020-0180

**Published:** 2021-08-25

**Authors:** Yue Wang, Bingbing Wu, Shengrong Long, QiangLiu, Guangyu Li

**Affiliations:** Department of Neurosurgery, Weifang People’s Hospital, Weifang, China; Department of Neurosurgery, First Affiliated Hospital of China Medical University, Shenyang 110001, China; Department of Neurosurgery, Cancer Hospital of China Medical University, Liaoning Cancer Hospital & Institute, Shenyang, China

**Keywords:** glioma, hypoxia, WNK3, invasiveness

## Abstract

**Background:**

The primary features of malignant glioma include high rates of mortality and recurrence, uncontrollable invasiveness, strong angiogenesis, and widespread hypoxia. The hypoxic microenvironment is an important factor affecting the malignant progression of glioma. However, the molecular mechanisms underlying glioma adaption in hypoxic microenvironments are poorly understood.

**Objective:**

The work presented in this paper focuses on the role of WNK3 gene in glioma invasion under hypoxic conditions. Furthermore, we aim to explore its role in epithelial-to-mesenchymal transition (EMT).

**Methods:**

ShRNA targeting WNK3 transfection was used to knockdown the WNK3 expression in U87 cells. We used western blot analysis to detect the relative expression of proteins in U87 cells. The effect of WNK3 on cell migration was explored using a transwell assay in the U87 cell line. We also evaluated WNK3 expression levels in glioma samples by immunohistochemistry analysis.

**Results:**

WNK3 expression was significantly higher in high-grade (III and IV) gliomas than in low-grade (I and II) gliomas. WNK3 expression was up-regulated in U87 cells when cultured in a hypoxic environment in addition; WNK3 knockdown inhibited the invasion of U87 glioma cells by regulating the EMT, especially under hypoxic conditions.

**Conclusion:**

These findings suggested that WNK3 plays an important role in the hypoxic microenvironment of glioma and might also be a candidate for therapeutic application in the treatment of glioma.

## 1 Introduction

Glioblastoma multiforme (GBM) is the most common and the deadliest type of malignant brain tumor in adults, also the most malignant tumor of glioma [1]. Despite numerous studies focus on GBM, patients with GBM still have a short period of survival time [2]. GBM often exhibits highly invasive growth, which contributes to poor outcomes in glioma patients [3,4]. GBM is characterized by intratumoral hypoxia caused by insufficient blood supply [5,6], which is a major feature of solid tumors and is involved in the regulation of tumor invasion [7,8]. Additionally, uncontrolled hypoxia leads to the formation of necrotic areas [9,10]; pseudopalisades, hypercellular zones near necrotic foci, are observed in GBM [11]. These cells migrated from the hypoxic focus, with 5–50% decrease in proliferation and 20–60% increase in cell migration. Therefore, in glioma, hypoxia correlates with the high invasive ability of tumors. Epithelial-to-mesenchymal transition (EMT) is a specialized cellular program that drives plasticity during wound healing, embryogenesis, and malignant progression [12]. An important mechanism of hypoxia-induced invasive ability is the stimulation of the EMT [8,13]. The EMT involves morphological changes from epithelial to mesenchymal phenotypes and enhances metastasis and invasiveness [14]. In tumors, the EMT includes the detachment of tumor cells from the basement membrane and facilitates the invasive capabilities of epithelial tumor cells without diminishing their viability.

With-No-Lysine Kinase (WNK) is a member of the serine/threonine-protein kinase family; it is named for the absence of a conserved lysine within the kinase domain. The WNK family comprises four human genes, WNK1–4 [15]. While most research has focused on the role of WNKs in hypertension [16], a growing body of evidence shows that WNKs are involved in various cancers, including glioma and other brain tumors [17]. Cancer stem cells (CSCs) have been found in a wide range of human tumors, although CSCs are a small fraction of tumor cells, which constitute the origin and development of various malignant tumors, and can serve as a key link in the process of tumor metastasis and recurrence [18]. A recent study suggested that WNK3 stimulates glioma invasion by regulating cell volume through the WNK3-NKCC1 pathway [19]. However, little is known about the role WNK3 plays in the hypoxic response of glioma and its impact on the EMT.

In the present study, we discovered that WNK3 was up-regulated during hypoxic conditions in the U87 glioma cell line. Importantly, we found that WNK3 can promote glioma invasion by regulating the EMT process. These data illuminated the role of WNK3 in the hypoxic response in glioma cells and suggested that WNK3 may serve as a new potential target of therapy for GBM.

## 2 Materials and methods

### 2.1 Cell culture

The human glioma cell lines U87, U251, and U373 were obtained from the American Type Culture Collection (ATCC, Manassas, VA, USA). Cells were cultured in DMEM (Gibco, USA) supplemented with 10% fetal bovine serum (Gibco, USA) and penicillin/streptomycin (100 U/mL). Cells were cultured under hypoxia (1% O_2_, 5% CO_2_, and 94% N_2_) and normoxia (5% CO_2_, and 95% O_2_) at 37°C.

### 2.2 Cell invasion assays

Cell invasion assays were performed in 24-well plates using Transwell invasion chambers with 8 m pore diameter polycarbonate (Corning Incorporated, Corning, NY, USA). Cells were seeded on the top side of the membrane, which was pre-coated with Matrigel (BD, Franklin Lakes, NJ, USA) in DMEM containing 0.2% serum (Gibco, USA). The lower chamber was filled with DMEM containing 20% serum. After incubation at 37°C for 20 h under hypoxic or normoxic conditions, non-invasive cells in the upper chamber were removed by wiping. The cells on the lower chamber surface (invasive cells) were stained with 0.1% crystal violet for 30 min and washed three times in phosphate buffer saline (PBS). The invasive cells were counted six random horizons in each well using a 10× objective.

#### 2.2.1 Immunohistochemistry analysis

Sixty-three samples of GBM tissues were selected from the First Affiliated Hospital of China Medical University. Through the use of immunohistochemistry, expression of WNK3 was detected in the 6-μm-thick paraffin-embedded GBM tissues. The primary antibody used was WNK3 (1:150; Abcam, UK). We used the DAKO Envision kit to stain and view samples according to the manufacturer’s instructions (DAKO, CA). Slides were photographed using an optical microscope (Olympus). The immunohistochemical staining intensity scores were as follows: two representative high-magnification fields were selected in each specimen, counting the average number of the 200 glioma cells. Positive cells <5% for the negative (−), 5–50% as weak positive (+), and >50% strongly positive (++).

**Ethical approval:** The research related to human use has been complied with all the relevant national regulations, institutional policies and in accordance the tenets of the Helsinki Declaration, and has been approved by the ethics committee of First Hospital of China Medical University (No. 2017-98-2).**Informed consent:** Informed consent has been obtained from all individuals included in this study.

#### 2.2.2 Plasmid extraction

(1) Add 200 μL of the original bacteria solution to 5 mL LB culture broth +5 μ ampicillin and place it on a shaker for 12–16 h. (2) 5 mL of bacterial solution, use 4.3 mL, add the remaining 0.7 mL to the cryopreservation solution (prepared with glycerol and LB culture solution 1:1) repeatedly and gently mix, then put it in the refrigerator at −80°C. (3) Prepare the extraction plasmid tube, put the adsorption sieve into the collection tube and add 500 μL of balance solution BL, centrifuge at 12,000 rpm for 1 min, discard the supernatant in the adsorption sieve, aspirate the plasmid tube bacteria solution, and save the lower layer bacteria. (4) Add 250 μL of solution P1 to the bacterial tube and suspend by pipetting. (5) Add 250 μL P2, turn up and down 6–8 times within 5 min, and mix well until it is clear. (6) Add 350 μL P3, turn up and down 6–8 times, centrifuge at 2,000 rpm for 10 min after a white precipitate appears. (7) Heat distilled water to 60°C, add distilled water to the supernatant in the suction tube, and centrifuge at 12,000 rpm for 1 min. (8) Pour out the liquid at the bottom of the sieve tube, add 500 μL protein solution PD, centrifuge at 12,000 rpm for 1 min, and pour out the lower layer liquid. (9) Add 600 μL of PW, centrifuge at 12,000 rpm for 1 min, pour out the lower layer of liquid, and repeat the process. (10) After centrifugation at 12,000 rpm for 2 min, throw away the lower layer of liquid, put the upper layer in a new 1.5 mL EP tube, and open the upper layer to volatile the liquid. (11) Add 30–50 μL of distilled water at 60°C to the upper layer. Be careful not to mix it and leave it at room temperature for 2 min. Centrifuge at 12,000 rpm for 2 min. (12) The liquid from the lower layer was sucked out and dropped into the upper layer, and the extraction was completed after centrifugation at 12,000 rpm for 2 min.

#### 2.2.3 Plasmid transfections

(1) U87 cells were inoculated in 6-well plates at a density of 2 × 10^5^/well. (2) The next day, the cells were observed under a microscope and transfected when they grew to 80%. (3) Use PBS for cell exchange, add 10 μL LP2000 and 3 μg plasmid (WNK3 shRNA and control shRNA) to 100 μL serum-free OPTI-MEM, mix and let stand for 5 min. (4) Mix the above two mixed liquids gently and stand for 20 min, add 1,800 μL of serum-free OPTI-MEM, and then add to the corresponding cell wells. (5) Change the liquid after 4–6 h. (6) Perform biological behavior analysis 24 h after transfection or collect cell extract protein after 48 h.

### 2.3 ShRNA and control stable cell lines

The U87 human glioma cell line was transfected with shRNA targeting WNK3 (WNK3 shRNA; Genechem, Shanghai, China). The control group was established using the same method and non-targeting shRNA (control shRNA; Genechem, Shanghai, China). The shRNA reagent transfection procedures were performed in accordance with the manufacturer’s instructions (Invitrogen; Carlsbad, CA, USA). The level of knockdown that shRNA achieved was assessed by western blot in all experiments.

### 2.4 Western blot

Total protein was extracted from cells, and the concentration was determined using the BAC protein concentration assay kit (Beyotime Biotec, China). Samples were separated using 8–15% SDS-PAGE and then transferred to PVDF membranes (Millipore, NY, and the USA). Membranes were blocked for 1 h with 5% non-fat milk. Membranes were incubated with primary antibodies at 4°C. Antibodies used in this study were as follows: rabbit anti-human WNK3 (1:2,000; Abcam, UK), rabbit anti-human Cyclin D1 (1:2,000, Cell Signaling Tech), rabbit anti-human MMP-2 (1:2,000, Abcam), rabbit anti-human MMP-9 (1:2,000, Abcam), rabbit anti-human Snail1 (1:2,000, Abcam), rabbit anti-human E-cadherin (1:3,000, Abcam), rabbit anti-human Vimentin (1:3,000, Abcam), and mouse anti-human β-actin (1:2,000, Abcam). After three washes in TBST, the membranes were incubated with the appropriate secondary antibody (1:5,000, Abcam) in TBST for 2 h at room temperature. Proteins were detected using the ECL detection solution (Apexbio, Houston, USA).

### 2.5 Statistical analysis

SPSS20.0 statistical analysis software was used to analyze the experimental data. The student’s *t*-test was used to compare data between two groups (two-tailed, unequal variance). Data were presented as the mean ± SD from a minimum of three independent experiments performed in triplicate. The Pearson’s chi-square test was used to analyze the relationship between WNK3 expression and the pathological grade of the glioma. Differences were considered significant when *P* < 0.05.

## 3 Results

### 3.1 WNK3 expression in human glioma tissue and cell lines

**Figure 1:**
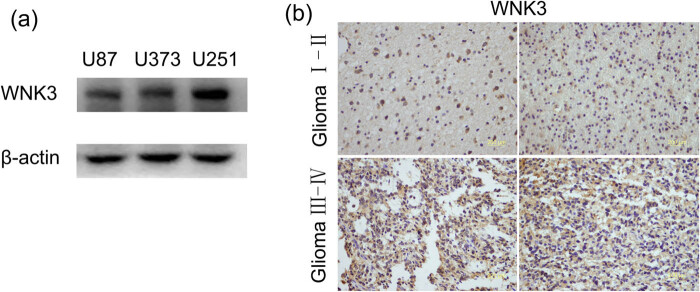
WNK3 immunohistochemistry (IHC) in glioma tissues. (a) The expression of WNK3 in U87, U373, and U251 glioma cell lines was detected by Western blot. (b) IHC staining for WNK3 in glioma stage I–II and Ⅲ–Ⅳ tissues. All photomicrographs were taken at 200× magnification; hematoxylin counterstain.

To investigate if WNK3 was expressed in glioma cells, we first assessed three widely used glioma cell lines: U87, U373, and U251 (Figure 1a). At the same time, the expression levels of WNK3 in the NBT (Normal Brain Tissue) and GBM tissues of the two data sets TCGA and REMBRANT were analyzed (Figure S4). In addition, we used immunohistochemical staining to evaluate the expression of WNK3 in 63 glioma tissue samples. We found that WNK3 was expressed in different grades of glioma tissues (Figure 1b). Furthermore, we found that WNK3 expression was more prevalent in high-grade (III and IV) gliomas than in low-grade (I and II) gliomas (Table 1). The expression level of WNK3 in the NBT and GBM tissues of the two data sets TCGA and REMBRANT.

**Table 1:** WNK3 expression in glioma tissue with different grades

	Case	WNK3 protein expression		*P*-value
	(63)	Weak	Strong	
WHO classification of glioma				
Grade I–II	26	21	5	*P* < 0.001***
Grade III–IV	37	9	28	

***Compared with Grade I–II and Grade III–IV, the high-grade gliomas have more positive expression of WNK3 than low-grade gliomas.

### 3.2 Expression of WNK3 is up-regulated in the U87 and U373 cell line in hypoxic conditions

**Figure 2:**
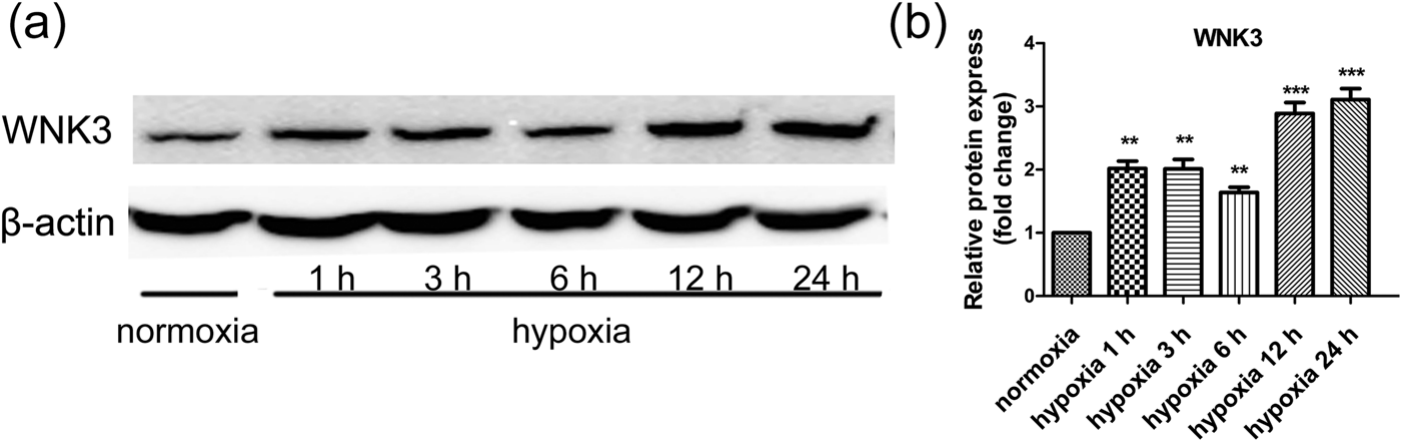
The expression of WNK3 in U87 significantly increased under hypoxia. (a) Cells were cultured under normoxic and hypoxic conditions (1, 3, 6, 12, and 24 h). Extracts from each lysate were subjected to western blot analysis. Western blot results showed the levels of WNK3 in U87 cells, and β-actin was used as an internal loading control. (b) Quantitative analysis of relative protein expression. Data were expressed as mean ± SD. All experiments were repeated three times (*P < 0.05; **P < 0.01; ***P < 0.001 vs the control group).

Western blot analysis was used to investigate WNK3 expression in the U87 and U373 cell lines in hypoxic and normoxic conditions. Cells were cultured in normoxia and hypoxia (1% O_2_ 1, 3, 6, 12, and 24 h), and WNK3 expression was measured (Figure 2a and Figure S2). WNK3 expression was up-regulated in U87 cells cultured in hypoxic conditions (Figure 2b), and the highest level of WNK3 expression was observed after 24 h of growth in hypoxic culture (*P* < 0.001).

### 3.3 WNK3 is involved in the invasion of U87 and U373 cells in hypoxic culture

**Figure 3:**
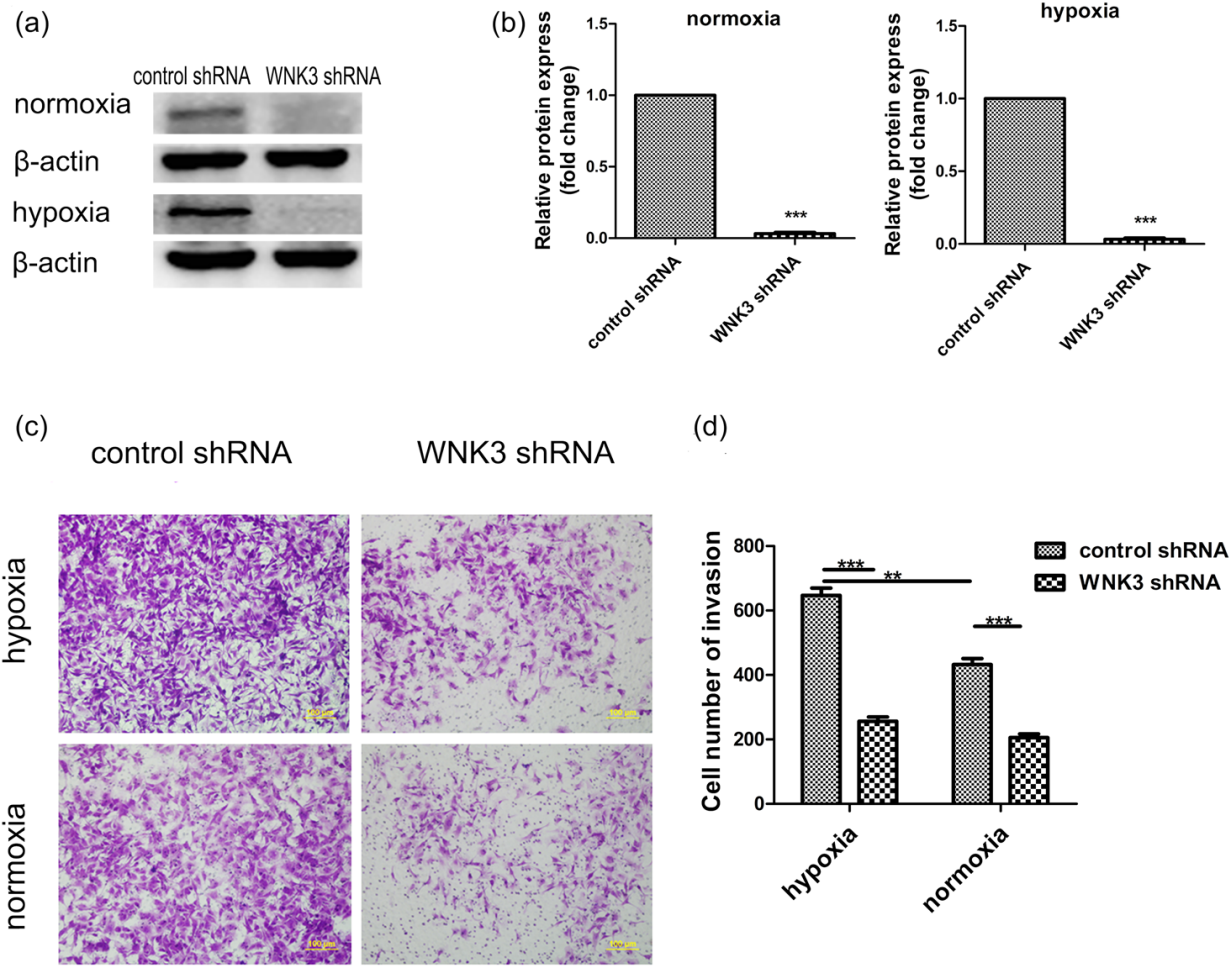
WNK3 Knockdown significantly inhibits the invasion of hypoxic U87 cells. (a) Western blot results showed the expression levels of WNK3 protein in U87 cells after transfection with shRNA, β-actin was used as an internal loading control. (b) Relative protein expression of results presented in (a) confirmed that WNK3 was knocked down. (c) Crystal violet staining of migratory U87 cells to measure invasiveness (400× magnification). (d) The number of cells that crossed the Transwell invasion chamber in normoxic and hypoxic conditions. The graph showed the mean ± SD and *P < 0.05, **P < 0.01, and ***P < 0.001.

To determine the role of WNK3 in the invasion of U87 and U373 cells, we constructed and verified short hairpin (sh)RNA targeting WNK3 (Figure 3a and b). We measured cell invasion by Transwell assays after transfected plasmids containing the shRNA into U87 cells. Tumor cell invasion through the extracellular matrix is an important step in tumor metastasis. We used Matrigel to serve as a reconstituted basement membrane matrix and counted the number of cells that migrated through the Matrigel matrix. Compared with control shRNA U87 cells, WNK3 shRNA U87 cells showed significantly decreased levels of invasion (432.7 ± 28.27 vs 205.7 ± 17.26, Figure 3c and d) when grown in normoxic conditions. The same results were observed when comparing control shRNA U373 cells with WNK3 shRNA U373 cells when grown in normoxic conditions (204.0 ± 31.00 vs 120.0 ± 22.00, Figure S1a and b). We performed the same experiment under hypoxic conditions and found that the invasive ability of U87 and U373 cells was significantly enhanced (Figure 3d and Figure S1b). Furthermore, WNK3 knockdown resulted in a more significant decrease in the level of invasion in hypoxic growth conditions (646.7 ± 32.93 vs 252.3 ± 19.37, Figure 3d and 271.6 ± 29.66 vs 133.3 ± 19.33, Figure S1b). These results indicated that inhibition of WNK3 expression significantly reduced the invasion of U87 and U373 cells.

### 3.4 WNK3 promotes hypoxia-induced EMT transition in U87 and U373 cells

**Figure 4:**
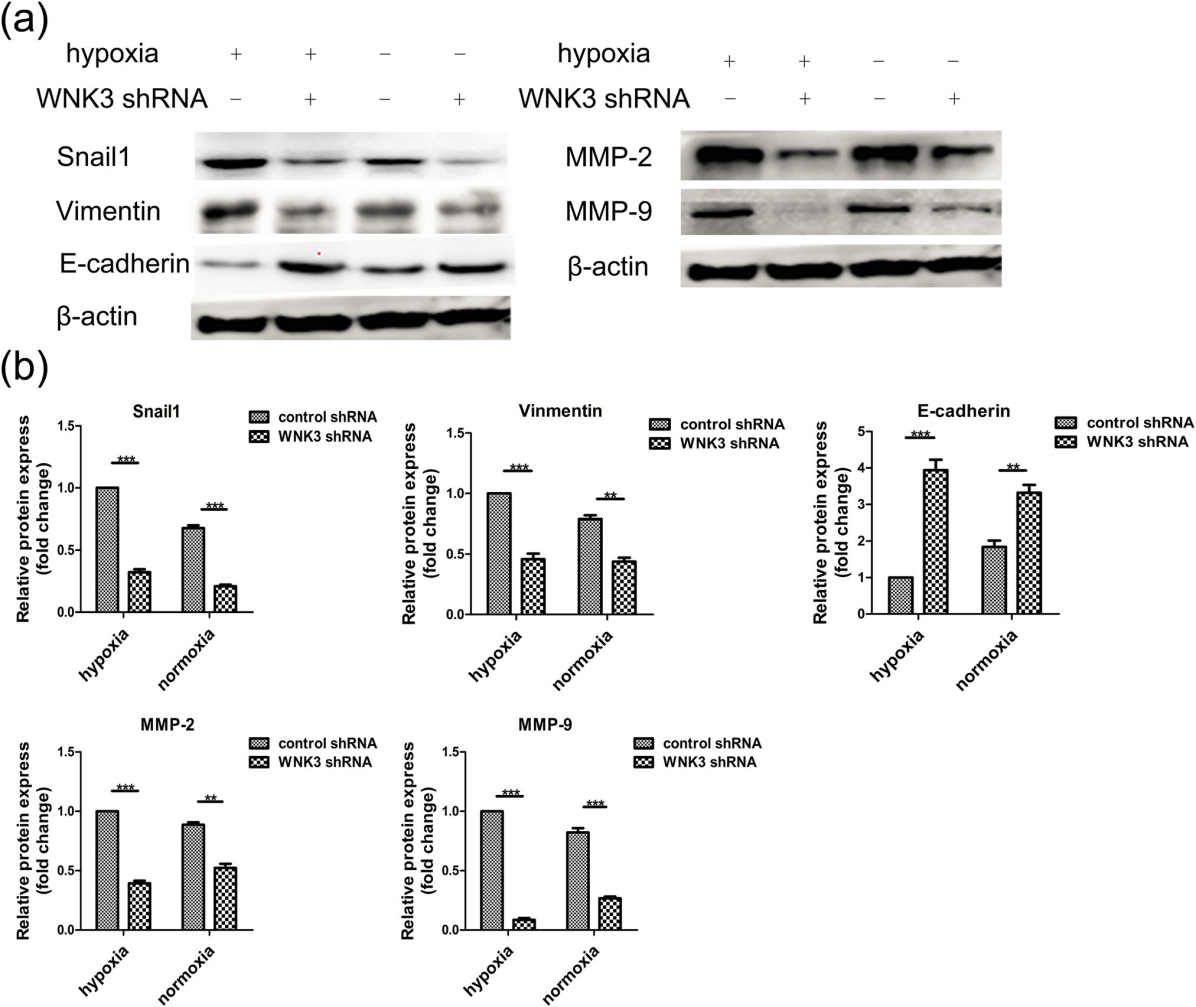
WNK3 knockdown significantly affects the expression of MMPs and EMT-related proteins in hypoxia. (a) Western blot analysis of WNK3 knockdown U87 cells showed the expression levels of Snail1, Vimentin, and E-cadherin and invasion-related proteins MMP-2 and MMP-9. β-Actin was used as an internal loading control. (b) Quantitative analysis of the EMT-related proteins Snail1, Vimentin, and E-cadherin and invasion-related proteins MMP-2 and MMP-9. Data are expressed as mean ± SD. All experiment.

To further investigate the role of WNK3 in U87 and U373 cell invasion, we measured the expression of the EMT-promoting proteins Snail1 and Vimentin in control and transfected U87 and U373 cells (Figure 4a and Figure S3). The levels of Snail1 and Vimentin proteins significantly decreased after WNK3 knockdown in U87 and U373 cells under normoxic (*P* < 0.01) and hypoxic (*P* < 0.001) conditions (Figure 4a and b). More importantly, in control shRNA U87 cells, the levels of Snail1 and Vimentin expression were higher when cells were cultured in hypoxic conditions than when grown in normoxic conditions (Figure 4). Furthermore, we measured E-cadherin, a marker reported to be down-regulated in the EMT process. The expression pattern of E-cadherin was the opposite of that observed for the EMT-promoting proteins Snail and Vimentin. These experiments indicated that WNK3 may promote EMT of U87 cells under hypoxic and normoxic conditions. We also measured the expression of the invasion-associated molecules MMP-2 and MMP-9 (Figure 4) and found that the expression levels of these proteins decreased upon down-regulation of WNK3. These results indicated that during hypoxia WNK3 promoted the invasion of U87 cells by inducing EMT.

### 3.5 WNK3 influences glioma cells growth *in vitro*

**Figure 5:**
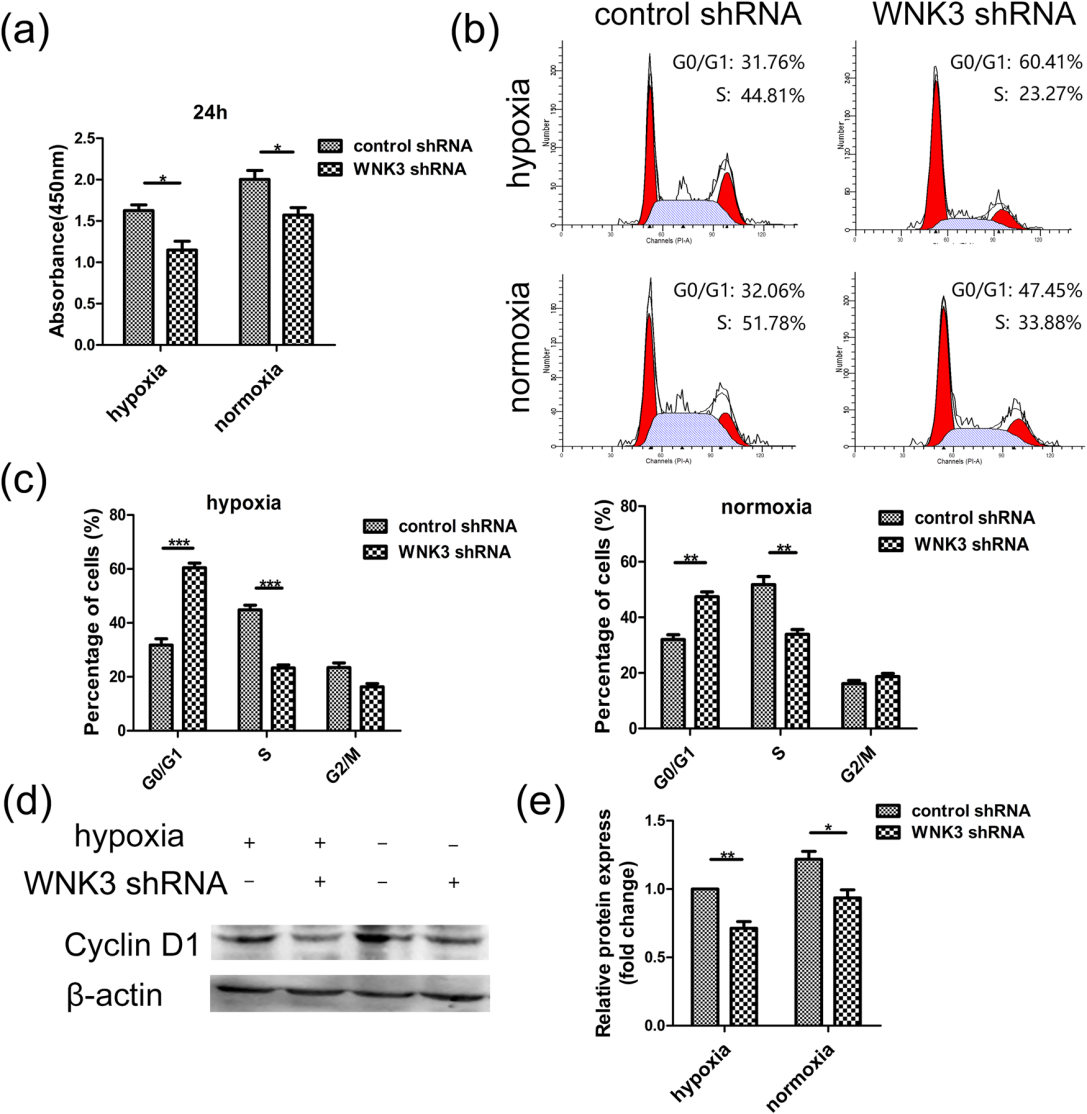
WNK3 knockdown inhibits U87 cell proliferation in hypoxic and normoxic conditions. (a) CCK-8 assay results showed that knockdown of WNK3 influences the growth of U87 cells in hypoxia and normoxia. (b and c) Cell cycle analysis evaluated the effects of WNK3 knockdown on cell cycle distribution in U87 cells during hypoxia and normoxia. (d and e) CyclinD1 expression in WNK3 shRNA U87 cells and control cells under hypoxia and normoxia, β-actin was used as an internal loading control. Data were expressed as mean ± SD. All experiments were repeated three times (*P < 0.05; **P < 0.01; ***P < 0.001).

We investigated whether WNK3 knockdown influences the growth of U87 cells by CCK-8 assay (Figure 5a). The CCK-8 assay showed that knockdown of WNK3 reduced the proliferation of U87 cells under normoxic and hypoxic conditions (*P* < 0.05). Therefore, we further examined the growth of U87 cells using flow cytometry (Figure 5b). We found that WNK3 knockdown induced cell cycle arrest and increased the percentage of cells in the G0/G1 phase while decreasing the percentage of cells in the S phase in U87 cells under hypoxic and normoxic conditions (Figure 5b and c). To explore the mechanism by which WNK3 regulates glioma cell proliferation, we measured the expression of the proliferation-associated protein CyclinD1 (Figure 5d). We observed that CyclinD1 protein levels were significantly down-regulated in WNK3 knockdown U87 cells under normoxic and hypoxic conditions (*P* < 0.05). These results suggested that inhibition of WNK3 expression can inhibit U87 cell proliferation by inducing cell cycle arrest in both normoxic or hypoxic conditions.

## 4 Discussion

In this study, we demonstrated that hypoxia can up-regulate WNK3 expression in U87 glioma cells. U87 cells grown in hypoxic conditions had enhanced invasive abilities through inducing EMT. Knockdown of WNK3, by shRNA, partially abolished the increased invasiveness during hypoxia and partially inhibited the proliferation of U87 cells.

Solid tumors have a wide range of cell hypoxia and ischemia, in part due to their rapid growth and relative lack of angiogenesis [20]. Indeed, glioma is no exception, and the majority of glioma cells have levels of hypoxia ranging from 0.1–10% PO_2_, with the average PO_2_ being 1% [7]. Furthermore, hypoxia is an important prognostic marker in glioma [21]. Hypoxic microenvironments can promote glioma transformation into a more malignant phenotype by enhancing invasiveness [9,22]. Recent reports suggested that WNK3 stimulates glioma invasion by regulating cell volume [19], meanwhile another study implied that WNK3 plays important role in the response of ischemia-induced brain damage [23]. It is reasonable for us to speculate that WNK3 may also be involved in enhancing the invasiveness of glioma in hypoxic conditions. Our results revealed that WNK3 expression was more prominent in high-grade glioma than low-grade glioma. Accordingly, it has been reported that hypoxia was more common and serious in high-grade glioma than in low-grade glioma [7]. Furthermore, we measured WNK3 expression and found that WNK3 expression was elevated in hypoxic conditions. All these data testified the speculation that WNK3 was involved in the hypoxic response in glioma cells. To explore the mechanism of hypoxic regulation in WNK3, we analyzed the promoter region of WNK3 and found a putative hypoxia response element (HRE) at the −187 bp upstream of the transcript start site, also we found repetitive AC sequence nearby the putative HRE (−300 bp upstream of the transcript start site), which is considered to be essential for HRE function. HRE is the binding site for hypoxia-inducible factor-1α (HIF-1α), the key regulator of the hypoxic response [24]. So, HRE of the WNK3 promoter should be identified in the future.

Although hypoxia can promote glioma invasiveness, the exact mechanism of how glioma cells invade the surrounding tissue is yet fully elucidated. EMT is one of the most studied mechanisms that confer to glioma cells this invasive capacity [25]. Some researchers overviewed that the reverse process of EMT and mesenchymal-to-epithelial transition (MET) must be key features of metastatic-initiating cells or “metastatic stem cells” to disseminate, extravasate, and be able to form colonies at the metastatic target organ site [26]. EMT is activated and regulated by specific micro-environmental endogenous triggers and complex signal pathway networks. These mainly include epigenetic events affecting protein translation-controlling factors and proteases, which are coordinated by the switches of oncogenes and tumor suppressor genes in cancer cells [27]. The regulation of EMT is an extremely complex, multiple pathways, such as transforming growth factor β (TGF-b), Wnt–β-catenin, Hedgehog， bone morphogenetic protein (BMP), Notch, and receptor tyrosine kinases, was reported to participant in this process [28]. Many molecules are involved in this process via these pathways [29,30]. To the best of our knowledge, no report about the role of WNK3 in the EMT process, especially under hypoxic conditions, is available, although WNK3 has been reported to promote invasiveness in glioma cells. Our data confirmed that WNK3 was involved in hypoxia-induced EMT and consequently regulate glioma invasive. The precise mechanisms by which WNK3 regulates the EMT in glioma are not clear. The most studied pathway of WNKs is WNKs-SPAK/OSR1-NKCC1 pathway [31], and to date, most of the researches about the effects of the WNK family on glioma invasion have focused on this pathway. NKCC1 can modify cell volume by regulating Cl^−^ concentration [32,33], and cell volume change is related to the EMT [34]. Therefore, WNK3 may influence the EMT through NKCC1. In addition, due to their large size and complicated structure, WNKs are regarded as atypical protein kinases with pleiotropic actions. Thus, other pathways, such as cross-talk with the TGF-β pathway, cannot be excluded. Indeed, WNK1 can interact with Smad2, a key protein in the TGF-β pathway [35]. Moreover, the TGF-β pathway is one of the most important pathways involved in the EMT [28]; further investigation into the mechanisms by which WNK3 influences the EMT in glioma should involve examination of these pathways. Our results were *in vitro* experiments. However, we are also eager to do *in vivo* experiments on animals in the near future, which cannot be completed at present due to the COVID-19 outbreak and the incomplete conditions for animal experiments, but we will continue to do further research in this field.

## 5 Conclusion

The results of the experiment indicated that WNK3 played an important role in the hypoxic response of glioma cells and exerted a widespread influence on the invasion of glioma cells during both normoxia and hypoxia. Thus, WNK3 kinase might serve as a novel therapeutic target for the treatment of these malignant brain tumors.
